# Impact of pre-operative antimicrobial treatment on microbiological findings from endocardial specimens in infective endocarditis

**DOI:** 10.1007/s10096-018-03451-5

**Published:** 2019-01-24

**Authors:** Mika Halavaara, Timi Martelius, Asko Järvinen, Jenni Antikainen, Pentti Kuusela, Ulla-Stina Salminen, Veli-Jukka Anttila

**Affiliations:** 1 Department of Infectious Diseases, Inflammation Center, Helsinki University Hospital (HUH) and University of Helsinki, Meilahti Triangle Hospital, open-plan office, 1st floor, P.O. BOX 372, 00029 Helsinki, Finland; 2 Division of Clinical Microbiology, HUSLAB, Helsinki University Hospital and University of Helsinki, Helsinki, Finland; 3 Department of Cardiac Surgery, Heart and Lung Center, Helsinki University Hospital and University of Helsinki, Helsinki, Finland

**Keywords:** Endocarditis, 16s rRNA PCR, Blood culture negative endocarditis, Valve culture, Valve surgery

## Abstract

Treatment of infective endocarditis (IE) should be initiated promptly. This might hamper the chances to identify the causative organism in blood cultures. Microbiological sampling of infected valve in patients undergoing surgery might identify the causative organism. The impact of pre-operative antimicrobial treatment on the yield of valve samples is not known. This study evaluated the impact of the duration of the pre-operative antibiotic treatment on valve culture and 16S rRNA PCR findings from resected endocardial samples. Patients meeting the modified Duke criteria of definite or possible IE and undergoing valve surgery due to IE during 2011–2016 were included from Southern Finland. Eighty-seven patients were included. In patients with shorter than 2 weeks of pre-operative antimicrobial treatment, PCR was positive in 91% (*n* = 42/46) and valve culture in 41% (*n* = 19/46) of cases. However, in patients who had 2 weeks or longer therapy before operation, PCR was positive in 53% (*n* = 18/34) and all valve cultures were negative. In 14% of patients, PCR had a diagnostic impact. In blood-culture negative cases (*n* = 13), PCR could detect the causative organism in ten patients (77%). These included five cases of *Bartonella quintana*, one *Tropheryma whipplei*, and one *Coxiella burnetii*. Long pre-operative antimicrobial treatment was shown to have a negative impact on microbiological tests done on resected endocardial material. After 2 weeks of therapy, all valve cultures were negative, but PCR was positive in half of the cases. PCR aided in diagnostic work-up, especially in blood culture negative cases.

**Electronic supplementary material:**

The online version of this article (10.1007/s10096-018-03451-5) contains supplementary material, which is available to authorized users.

## Introduction

Treatment of infective endocarditis (IE) is challenging. It demands skills from different medical and surgical specialties and necessitates promptly initiated and prolonged intravenous antimicrobial therapy [1, 2]. Surgery is needed in almost half of the cases [1]. An important prerequisite for optimal antibiotic therapy, and hence to a better prognosis, is identification of the causative agent. In most cases, this is acquired from blood cultures. In blood-culture negative cases, etiology may be revealed by serological testing (e.g., *Bartonella quintana* or *Coxiella burnetii* antibody assay) or, in patients undergoing valve surgery, by microbiological and molecular testing done on resected heart valves [3, 4]. Of molecular methods, broad range PCR has proven to be a useful diagnostic tool and more sensitive than valve culture [5, 6]. However, the effect of antimicrobial treatment duration on diagnostic yield of valve culture or PCR has not been characterized [6–11].

We collected data on all surgically treated patients with IE during 6 years from a center taking care of a population of 1.9 million to study the impact of length of pre-operative antibiotic treatment on the diagnostic yield of valve cultures and broad range bacterial 16S rRNA PCR. Another aim was to evaluate the diagnostic impact of valve PCR and to gather information on the non-cultivable agents behind IE in Finland.

## Materials and methods

The study design was a retrospective single-center study. Patients (*n* = 115) who underwent cardiac surgery due to IE in Helsinki University Hospital (HUH) during 2011–2016 were identified from the operating room’s database using ICD-10 codes for infective endocarditis (I33, I38, and I39). The inclusion criteria were the following: (1) a sample for PCR analysis was obtained during surgery and (2) patients were post-operatively classified as having possible or definite IE according to modified Duke criteria [12]. The exclusion criteria were the following: (1) a sample for PCR analysis was not obtained during surgery (*n* = 22), (2) the data was not available (*n* = 2), (3) IE was caused by yeast (*n* = 2), and (4) IE diagnosis was rejected according to modified Duke criteria (*n* = 2). In all, 87 patients were included in the final analysis.

HUH is the sole provider of cardiac surgery in the Hospital District of Helsinki and Uusimaa and two smaller adjacent hospital districts in southern Finland. The population is 1.9 million and mainly urban, but rural areas are also included.

An infectious disease specialist (M.H.) reviewed all patient data from the medical and laboratory records. Demographic and clinical data were recorded.

### Study definitions

Effective antibiotic treatment was defined as follows: an antibiotic treatment to which the causative agent was susceptible to in vitro; an antibiotic treatment according to the European Society of Cardiology guideline [2]; and delivered intravenously. Duration of pre-operative treatment was calculated from the first day of receipt of intravenous effective antibiotic therapy to the operation day. Antibiotic therapy had to be continuous. If patient had received a period of antibiotic therapy previously, but it was discontinued more than 2 weeks before admission, it was not included into duration of preoperative treatment.

Patients were divided into five groups depending on the length of the pre-operative effective intravenous antibiotic therapy as shown in Table 1. The last group (*n* = 7) consisted of patients who received less than 1 week of intravenous therapy before surgery but had a longer continuous oral and intravenous treatment before it. In most cases, their longer course had targeted foci other than IE. Thus, this group is left out of the time analysis, because true length of effective therapy could not be determined.

**Table 1:** Impact of pre-operative antibiotic treatment on PCR results and valve cultures

Intravenous antibiotic treatment started in relation to surgery	Number of cases (*n* = 87)	PCR result *n* (%)	Valve culture positive *n* (%)
On operation day (or after)	11	10 (90.9)	4 (36.4)
Within 2–13 days before operation	35	32 (91.4)	15 (42.9)
Within 14–28 days before operation	17	10 (58.8)	0
More than 28 days before operation	17	8 (47.1)	0
Within 7 days before operation, but previous longer course	7	4 (57.1)	0

A result from PCR analysis was considered to have a diagnostic impact in the following situations: (1) it revealed etiology of IE when blood cultures were negative, (2) it confirmed positive serological tests, or (3) it resolved etiology of IE when there was a discrepancy between blood and valve cultures.

### Microbiological procedures

In HUH, microbiological and histological sampling is routinely done in operative treatment of patients with IE. Since 2010, surgeons have been advised to routinely include also a sample for PCR analysis along with other microbial samples (mainly bacterial cultures). Hence, this study evaluates the impact of PCR analysis implemented in routine clinical setting. From 87 patients included, 89 samples for PCR were obtained. In two cases, two samples were obtained and in the rest of the cases, one. In 80 cases, a sample for PCR was taken from the valve tissue or the vegetation and in 7 from pus, e.g., a paravalvular abscess. A total of 101 valve culture samples were obtained. One sample per patient was obtained in 73 cases and two samples in 14 cases. All valve culture and PCR results are presented per patient. These samples were sent immediately to laboratory (HUSLAB, Helsinki) for analysis. If necessary, samples were stored maximally for 2 days in +4 °C before analysis.

Blood cultures in cases with suspected IE were taken as routine protocol as ordered by the treating physicians. Blood samples were incubated in blood culture bottles in BacT/Alert® (BioMérieux, France) instrument a total of 5–6 days or until reported as positive, where after the microbe was identified by routine diagnostic methods.

The clinical samples were cultured by routine diagnostics at HUSLAB (Helsinki University Hospital Laboratory, Helsinki, Finland) using standard methods. Briefly, samples were cultivated on chocolate agar and fastidious anaerobe agar and incubated at 37 °C until growth was observed or for maximum 7 days in 5% CO_2_ or under anaerobic conditions, respectively. In addition, samples were also inoculated into the thioglycollate broth and cultivated at 37 °C.

For 16S rRNA analysis, the clinical samples were homogenized using a Precellys bead-beater (Bertin Instruments) and DNA was extracted with SelectNAplus (Molzym) instrument or previously with Easymag (BioMérieux) (see Supplement Table 1). Each method has been validated and no different effect on positivity rate has been observed. The ribosomal 16S DNA was amplified using primers Forward CLSI and Reverse Bosshard [13]. Used polymerases, PCR instruments, and thermal cycling conditions are specified in Supplement Table 1. PCR-amplified fragments were separated using gel electrophoresis and visualized under ultraviolet light. Amplified fragments were purified and sequenced with a BigDye Terminator v3.1 Cycle Sequencing Kit (Thermo Fisher Scientific) using forward Bosshard primer [13] and an ABI Prism 3100 genetic analyzer (Thermo Fisher Scientific). Then analysis was performed by Basic Local Alignment Search Tool (BLAST; National Center for Biotechnology Information, Bethesda, MD, USA. https://blast.ncbi.nlm.nih.gov/Blast.cgi). An inhibition control (amplification of lambda DNA or *Oryza sativa* gene) was tested with each sample and a nontemplate control was tested with each run.

### Statistical analysis

Categorical data was analyzed by chi-square test or Fisher’s exact test, as appropriate. Continuous variables were analyzed with Mann-Whitney *U* test. Statistical analysis was done using SPSS Software, version 22.0 (SPSS, Inc. Chicago, Illinois).

## Results

In total, 87 patients were included and their mean age was 52.7 years and 71 (81.6%) of them were male. Patient demographics and medical history are shown in Table 2. Data on valve surgery and clinical features are shown in Table 3. Definite IE was observed in 85 patients and 2 patients had possible IE according to modified Duke criteria [12].

**Table 2:** Demographics and medical history of the study cohort

Demographics (*n* = 87)	Age, years (mean)	52.7
	Male	71 (81.6%)
Medical history	Pre-disposing and pre-existing structural heart disease	24 (27.6)
	Previous IE	7 (8.0)
	Chronic kidney disease	4^a^ (4.6)
	Liver cirrhosis	4 (4.6)
	Diabetes	17 (19.5)
	Alcoholism	11 (12.6)
	IVDU	14 (16.1)

*IVDU* intravenous drug users

^a^3 hemodialysis, 1 peritoneal dialysis

**Table 3:** Valves operated due to IE and clinical data of study cohort

		Number of valves operated (*n* = 105)
Operated valve	Aortic	50
	Mitral	47
	Tricuspidal	7
	Pulmonal	1
		Number of patients (% of all)
Type of valve	Native	76 (87.4)
Cardiac device involved		2 (2.3)
Additional clinical data	Septic emboli (or deep focus)	48 (55.2)
	Relapse of IE within 12 months	4 (4.6)
	30-day all-cause mortality	7 (8)
	365-day all-cause mortality	15 (17.2)

### Blood and valve cultures

Blood cultures were positive in 74 (85%) cases (Table 4). Most common findings were *Staphylococcus aureus* (*n* = 24), *Streptococcus viridans* group (*n* = 22), and *Enterococcus faecalis* (*n* = 9).

**Table 4:** Results of blood cultures, valve cultures, and PCR samples. Number of patients with each microbe is shown

Microbe	Blood culture	Valve culture	PCR
Blood culture positive cases (*n* = 74)			
*Staphylococcus aureus*	25^a^	6	20
*Streptococcus viridans* group^b^	20	2	15
*Enterococcus faecalis*	9	2	4
Coagulase negative staphylococcus	8^c^	4^d^	4
*Streptococcus agalactiae*	4	1	4
Other beta-hemolytic streptococci	4^e^	0	3
*Streptococcus pneumoniae*	1	0	0
*Streptococcus bovis*	1	1	1
*Streptococcus anginosus*	1	1	1
*Granulicatella adiacens*	1	0	1
*Aerococcus urinae*	1	1	1
Blood culture negative cases, but PCR positive (*n* = 10)			
*Bartonella quintana*	0	0	5
*Coxiella burnetii*	0	0	1
*Tropheryma whipplei*	0	0	1
*Streptococcus pneumoniae*	0	0	1
*Streptococcus viridans* group	0	1	2
IE cases without determined etiology (*n* = 3)			

^a^In 2 cases, polymicrobial bacteremia (1 with *Bacillus cereus* and 1 with *Streptococcus agalactiae* and in this case *Staphylococcus aureus* was not considered as causative agent of IE)

^b^*Streptococcus anginosus and Streptococcus bovis* are shown separately

^c^4 *Staphylococcus epidermidis*, 3 *Staphylococcus lugdunensis*

^d^3 concordant with BC (same cases); 1 different case (ruled as contamination in that case)

^e^3 G group and 1 C group

Valve cultures were positive in 19 (22%) cases. In two cases, the valve culture result differed from the blood culture result. In one case, *Streptococcus mitis* did not grow on the blood cultures, but it was cultured from the valve and found in PCR analysis. In the other case, *Staphylococcus epidermidis* grew on the valve culture but *Streptococcus salivarius* was found in the blood cultures and valve PCR analysis. In this case, *Staphylococcus epidermidis* was regarded as a contaminant. Additionally, in one patient *Cutibacterium acnes* grew on culture. However, this sample was taken from a mediastinal site and blood cultures were positive for *Enterococcus faecalis*. Thus, this finding was ruled as a contaminant after infectious disease specialist’s consultation. In cases were two valve samples were taken, only in one case the results differed (one negative and the other positive).

### 16S rRNA PCR and sequencing

In 64 cases (74%), PCR analysis was positive (Table 4). Of these cases, 54 were also blood-culture positive with consistent results and 10 were blood culture negative. In only one case valve culture was positive (*Staphylococcus aureus*), but PCR remained negative. In blood-culture negative cases (*n* = 13), PCR analysis identified the causative agent in 10 (77%). In one case, a mixed sequence was suspected with *Streptococcus mitis* and possibly in addition other *Streptococcus viridans* group species. However, in this case, *Streptococcus mitis* grew on blood cultures. In two cases, two samples for PCR were taken. In both cases, the two samples were both positive with similar result (i.e., same bacteria).

We identified 12 cases where valve PCR was considered to have a diagnostic impact (Table 5).

**Table 5:** Cases in which PCR finding had a diagnostic impact (*n* = 12, 13.8%)

PCR finding	Number of cases	Type of impact	Comments
*Bartonella quintana*	5	Serological findings confirmed	Cultures negative; serology tested and positive in 3 cases
*Coxiella burnetii*	1	Serological findings confirmed	Serology positive; cultures negative
*Tropheryma whipplei*	1	Etiology of IE provided	No serological tests available and bacteria cannot be cultured
*Streptococcus gordonii*	1	Etiology of IE provided	Both valve and blood cultures negative; antibiotic treatment started before cultures
*Str. pneumoniae*	1	Etiology of IE provided	Both valve and blood cultures negative, antibiotic started before cultures
*Str. agalactiae*	1	Etiology of IE confirmed; discrepancy between valve and blood cultures	Valve culture negative; *Str. agalactiae* and *Staphylococcus aureus* both in blood cultures
*Str. salivarius*	1	As above	*Str. salivarius* in blood cultures; *Staphylococcus epidermidis* in valve culture^a^
*Str. mitis*	1	As above	Blood cultures negative; *Streptococcus mitis* in valve culture

*IE*, infective endocarditis; Str., streptococcus

^a^This culture result was ruled as a contaminant

### Impact of duration of antibiotic therapy on valve cultures and PCR findings

Patients were categorized according to the length of the pre-operative effective intravenous antibiotic therapy as shown in Table 1. After excluding the seven patients in whom the duration of effective antimicrobial therapy was uncertain (as described in detail in *Study definitions* paragraph), we chose to compare the groups with antibiotic duration less than 2 weeks with the groups with antibiotic duration more than 2 weeks. The proportion of PCR-positivity in those patients who received effective antimicrobial therapy less than 2 weeks before surgery was 91% (*n* = 42/46) and in patients who received it more than 2 weeks 53% (*n* = 18/34); *p* = 0.00009; chi-square.

To control for possible confounding, independent variables associated with PCR-positivity in univariate analysis (*p* value < 0.20 or clinical significance, Supplement Table 2) were included in a logistic regression analysis. Only duration of pre-operative antibiotic treatment less than 2 weeks remained as a significant denominator for PCR-positivity (OR 7.2, 95% CI 2.0–26.0; *p* = 0.003).

**Fig. 1:**
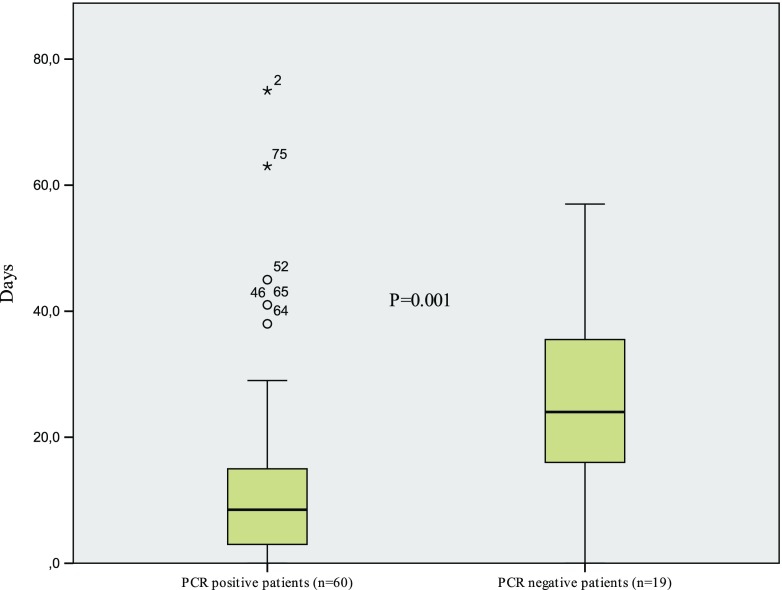
Duration of pre-operative antimicrobial therapy and PCR results. Bars show the median

The median duration of pre-operative antibiotic therapy in PCR-positive patients (*n* = 60) was 8.5 days and in PCR-negative (*n* = 19) 24.0 days, *p* = 0.001 (Fig. 1). One patient who received more than 4 weeks of pre-operative antibiotic treatment was additionally excluded from this median duration time analysis, because the exact duration of the antibiotic therapy could not be determined.

All valve culture positive patients (*n* = 19) received antibiotic therapy less than 2 weeks before surgery.

## Discussion

In this study, we observed that longer than 2 weeks of antimicrobial treatment totally abolished valve cultures and significantly reduced also the yield of valve bacterial PCR. However, even after 2 weeks of treatment PCR detected causative agent in more than half of the cases. 16S rRNA PCR contributed to the etiological diagnosis in 14% of cases. It showed benefit especially in blood culture negative cases in which 16S rRNA PCR could detect the causative agent in 77% of cases. In addition, we observed five cases of *Bartonella quintana* and one of *Coxiella burnetii* and *Tropheryma whipplei* each, which all have previously thought to be very rare in Northern Europe.

Effect of pre-operative antibiotic treatment on valve cultures is controversial, as two studies found no effect [6, 11], but two reported a negative effect [7, 9]. In our material, we had no positive valve cultures after 2 weeks of antimicrobial treatment. In accordance, only one valve culture was positive after 2 weeks of antibiotic treatment among 131 cases of streptococcal endocarditis [10].

In a study by Peeters et al. most (19/24) patients had a causative agent detected with 16S rRNA PCR even after 21 days of antibiotic therapy [6] and another study could not confirm any correlation between the length of pre-operative antibiotic treatment and positive PCR results [9]. Rovery et al. demonstrated that bacterial DNA may persist in heart valves for a long time after successful treatment and does not necessarily represent viable organisms [8]. However, in patients who underwent valve surgery while still on antibiotic treatment, bacterial DNA was detected significantly more often compared to patients who had completed the treatment. They concluded that bacterial DNA was cleared over time, but slowly. In the present study, 74% of patients had positive PCR results. In previous studies, the sensitivity of PCR ranged from 40 to 100% [5, 6]. However, after 2 weeks of effective intravenous antibiotic therapy, PCR positivity dropped markedly, but half were still positive. Our results suggest that in a real-life setup where PCR is included into routine operative procedures, pre-operative antimicrobial treatment seems also to reduce the yield of PCR. Furthermore, patients with a positive PCR test result had also shorter median duration of pre-operative antibiotic therapy than PCR negative cases.

Interestingly, in one case, PCR test could still detect bacterial DNA in the resected heart valve after 6 months of continuous antibiotic therapy. This patient had blood culture positive IE caused by *Granulicatella adiacens* and had a long intravenous antibiotic treatment initially. At the time of surgery, the patient was still on oral antibiotic therapy due to concomitant spondylodiscitis. After operation, the antibiotic therapy was discontinued and in follow-up, no relapse occurred.

One previous study from Finland evaluated 56 cases of IE requiring surgery and found that in 4 cases (7%) the causative agent was detected only by 16S rDNA PCR [7]. This study was done in a different region in Finland than our study and included the first published case of *Bartonella quintana* IE in Finland. A Swedish study including 57 patients found 16S rDNA PCR particularly useful in blood culture negative cases but included no cases due to *Bartonella* spp. [14]. In two studies from Denmark, including patients with IE undergoing surgery and molecular diagnostic methods, no cases of *Bartonella* spp. were reported either [9, 15]. Here, we report five cases of IE caused by *Bartonella quintana*, one caused by *Coxiella burnetii* (etiological agent in Q-fever) and one caused by *Tropheryma whipplei* which all have previously been reported mainly from Southern Europe. Furthermore, two more cases of IE caused by *Tropheryma whipplei* have been observed in our hospital outside the study period.

The present study adds to the weight of evidence of diagnostic value of PCR in surgically treated IE patients. In a Canadian study of 68 patients, molecular methods contributed to the microbial diagnosis of 31% of patients with IE requiring valve surgery and contributed to the clinical decisions in 13% [5]. Similarly, in a French study, valve PCR contributed to the microbiological etiology of IE in 20% of patients [4].

In this study, all bacteria found by PCR in the culture negative cases were organisms recognized to cause IE and not usually regarded as contaminants. In PCR negative cases, we cannot rule out the possibility of false-negative cases due to sampling errors. In some studies, there have been a high number of false-positive valve cultures raising a concern of their utility [8, 16]. However, in our study, only two valve cultures were ruled as contaminants.

The present study is from a single center which, however, is the sole provider of cardiac surgery in its recruitment area. Thus, we believe that virtually all patients from this area undergoing valve surgery for IE are included in this study as long as we have been able to identify the cases from the hospital records. Considering that infective endocarditis is a relatively rare condition that requires surgery in almost 50% of the episodes, the 87 patients of our study make it one of the largest of its kind. One of the main limitations of our study was its retrospective nature making it impossible to rule out a selection bias, especially when 22 patients were excluded because sample for PCR was not taken in spite of the hospital protocol. However, 12 of these occurred in year 2011 (the first year) and the remaining one to three cases annually. So, the most likely explanation is that surgeons had not yet adopted a new protocol rather than other systematic bias. Operating surgeons were instructed to take a sample for PCR from the vegetation or the visually infected valve tissue. Depending on the operative situation, the extent and intensity of the infection, the amount and the quality of the sample may have varied. More standardized sampling recommendations for PCR analysis might be needed.

In conclusion, 16S rRNA PCR was a valuable tool in etiological diagnosis of IE in patients undergoing valve surgery and especially in those with longer pre-operative antibiotic treatment and in blood-culture negative IE. Although the length of pre-operative antibiotic therapy had a negative effect on the yield of both valve cultures and PCR, after 2 weeks of antibiotic therapy, PCR was positive in nearly half of the patients. *Bartonella quintana*, *Coxiella burnetii*, and *Tropheryma whipplei* may present as causative agents also in the Northern Europe and seem to be found by valve PCR sampling.

## Electronic supplementary material

ESM 1 (XLSX 9 kb)

ESM 2 (DOCX 21 kb)
