# Supplementary material for: Impact of pre-operative antimicrobial treatment on microbiological findings from endocardial specimens in infective endocarditis
Source: Eur J Clin Microbiol Infect Dis. 2019 Jan 24;38(3):497–503. doi: 10.1007/s10096-018-03451-5 (PMC6394703; doi:10.1007/s10096-018-03451-5)
Supplement: Supplementary file 2 — (DOCX 21 kb) [file 10096_2018_3451_MOESM2_ESM.docx]

| Supplement Table 2. Difference in pre-operative antibiotic treatment duration, clinical features and sample quality of patients with PCR positivity and negativity in a group included in the time analysis (n=80) | | | |
| --- | --- | --- | --- |
| Variables | PCR positive (n=60) | PCR negative (n=20) | P-value^a^ |
| IV antibiotic treatment less  than two weeks^b^ | 42 (70) | 4 (20) | 0.0002 |
| Diabetes n (%)^b^ | 9 (15) | 6 (30) | 0.185 |
| Liver Cirrhosis | 3 (5) | 0 | 0.569 |
| Renal insufficiency | 2 (3.3) | 1 (5) | 1.000 |
| Hemodialysis | 2 (3.3) | 0 | 1.000 |
| Immunosuppression | 2 (3.3) | 1 (5) | 1.000 |
| Alcohol abuse | 7 (11.7) | 3 (15) | 0.705 |
| IVDU^c^ | 13 (21.7) | 0 | 0.031 |
| Native valve^b^ | 56 (93.3) | 14 (70) | 0.013 |
| PCR sample tissue^b^ | 56 (93.3) | 17 (85) | 0.358 |
| Number (column-%)  Abbreviations: IV, intravenous; IVDU, intravenous drug users  ^a^ Fisher´s exact test used to test the difference  ^b^ These variables included in logistic regression analysis  ^c^ Not included in logistic regression analysis, because number of IVDU PCR negative cases is zero | | | |
